# The effect and burden modification of heating on adult asthma hospitalizations in Shijiazhuang: a time-series analysis

**DOI:** 10.1186/s12931-019-1092-0

**Published:** 2019-06-14

**Authors:** Feifei Liu, Fangfang Qu, Huiran Zhang, Lingshan Chao, Rongqin Li, Fengxue Yu, Jitao Guan, Xixin Yan

**Affiliations:** The Second Hospital of Hebei Medical University, Shijiazhuang city, Hebei province China

**Keywords:** Ambient air pollutants, Asthma hospitalization, Heating season, Burden

## Abstract

**Background:**

Previous studies have found associations between asthma morbidity and air pollution especially in young population, (PLoS One 12:e0180522, 2017; Can J Public Health 103:4-8, 2012; Environ Health Perspect 118:449-57, 2010; Am J Respir Crit Care Med 182:307-16, 2010; J Allergy Clin Immunol 104:717-22, 2008; J Allergy Clin Immunol 104:717-22, 1999; Environ Res 111:1137-47, 2011) but most of them were conducted in areas with relatively low air pollutant level. Moreover, very few studies have investigated the effect and burden modification of heating season during which the ambient air pollution level is significantly different from that during non-heating season in north China.

**Objectives:**

This study aimed to evaluate the effect and burden modification of heating on short-term associations between adult asthma hospitalizations and ambient air pollution in the north China city of Shijiazhuang.

**Methods:**

Generalized additive models combined with penalized distributed lag nonlinear models were used to model associations between daily asthma hospitalizations and ambient air pollutants from 1 January 2013 to 16 December 2016 in Shijiazhuang city, adjusting for long-term and seasonality trend, day of week, statutory holiday, daily mean air pressure and temperature. Attributable risks were calculated to evaluate the burden of asthma hospitalizations due to air pollutants exposure. The effect of pollutants on hospitalization and the attributable measures were estimated in heating and non-heating season separately and the comparisons between the two seasons were conducted.

**Results:**

All pollutants demonstrated positive and significant impacts on asthma hospitalizations both in heating season and non-heating season, except for O_3_ in heating season where a negative association was observed. However, the differences of the pollutant-specific effects between the two seasons were not significant. SO_2_ and NO_2_ exposure were associated with the heaviest burden among all pollutants in heating season; meanwhile, PM_10_ and PM_2.5_ were associated with the heaviest burden in heating season.

**Conclusions:**

In conclusion, we found evidence of the effect of ambient air pollutants on asthma hospitalizations in Shijiazhuang. The central heating period could modify the effects in terms of attributable risks. The disease burden modification of heating should be taken into consideration when planning intervention measures to reduce the risk of asthma hospitalization.

## Introduction

Asthma is a well-known non-communicable disease with high morbidity, affecting over 334 million people around the world. Though this diagnosis can be linked to one’s genetics, it is important to recognize that asthma can be exacerbated by multiple preventable risk factors such as environmental pollution [1]. The adverse impact of ambient air pollution on asthma, especially in developing countries, is mostly attributed to rapid industrialization and increase in vehicle usage [2], and imposing a heavy social and economic burden on individuals, families, and countries.

Winter heating related fossil-fuel combustion has become a significant air quality and public health concern in north China. However, previous studies have traditionally taken the time-related season as a modifier [3]. It remains uncertain whether pollutants’ effects are modified by the central heating period covering winter and part of spring.

Furthermore, although consistent findings have indicated that ambient air pollutants played important roles in the exacerbation of asthma, resulting in increased emergency department visits [4, 5] and hospitalizations [6], the burden of asthma hospitalizations associated with the interaction between ambient air pollutants and heating has rarely been reported, particularly in a location of north China with extremely heavy air pollution such as Shijiazhuang city. Compared to the relative risk, the disease burden may provide more relevant information for policy-makers, help them comprehensively assess the adverse impacts of pollutants on asthma population and design different intervention strategies.

In the present study, we aimed to examine the modification effect of heating on the relationship between ambient air pollutants (including particulate matter (PM)with aerodynamic diameter < 10 μm(PM_10_) and < 2.5 μm(PM_2.5_), nitrogen dioxide (NO_2_), sulfur dioxide (SO_2_), carbon monoxide (CO) and ozone (O_3_)) and adult asthma hospitalizations in the urban area of Shijiazhuang from 2013 to 2016. We further estimated the morbidity burden in terms of attributable fraction (AF) and attributable number (AN) and evaluated their difference between heating and non-heating seasons.

## Material and methods

### Study area

**Fig. 1:**
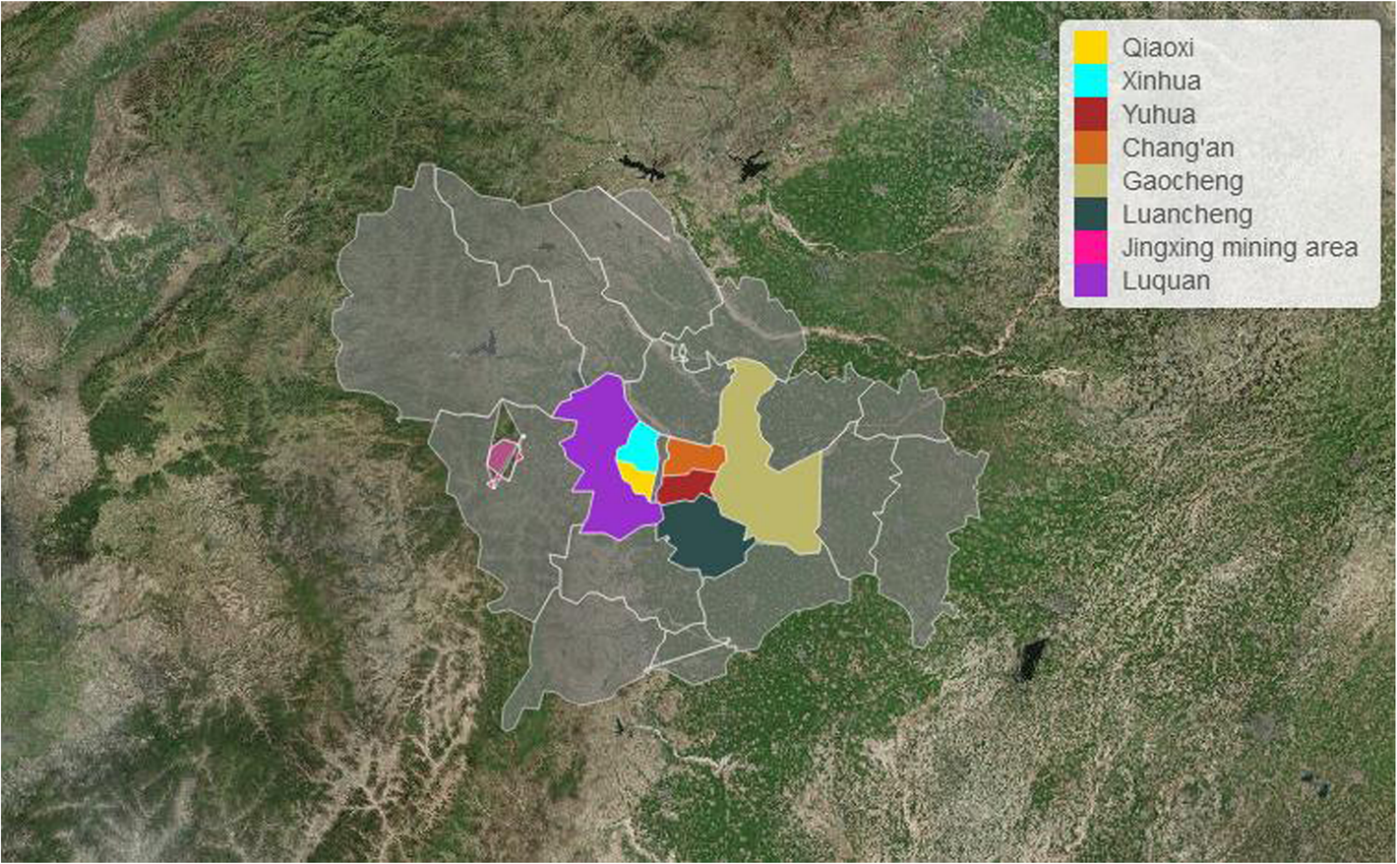
The geographical location and urban districts distribution of Shijiazhuang city

Shijiazhuang, the capital city of north China’s Hebei province, is located in north latitude 37° 27′~ 38° 47′ and east longitude 113° 30′ ~ 115° 20′, with the Taihang Mountains in the west and the vast north China plain in the east, south, and north. Due to temperate continental monsoon climate, ambient air pollutants over Shijiazhuang moved westward with the southeast wind. When blocked by the Taihang Mountains, they turned eastward and returned to the same place. Similarly, the northwest wind is intercepted in Shanxi province, so pollutants can only continue to stay in Shijiazhuang, resulting in continuous heavy pollution especially in winter [7]. Besides, as a mega-city, Shijiazhuang has over 4.81 million permanent residents in the urban area in 2016 with over 2.21 thousand km^2^, including eight districts named Xinhua, Qiaoxi, Chang’an, Yuhua, Jingxing, Gaocheng, Luancheng,and Luquan (Fig. 1).

### Study population

The data for asthma hospitalizations from 1 January 2013 to 16 December 2016 were obtained from the Medical Insurance Center of Shijiazhuang city, where covers about two-thirds of urban Shijiazhuang’s permanent residents and collects electronic hospitalization summary reports from the Electronic Medical Record System of all the tertiary hospitals and secondary hospitals in urban Shijiazhuang city. The criteria for data extraction in our study include: (1) a primary discharge diagnosis of asthma (International Classification of Diseases, ICD-9: J45.000, J45.003, J45.007, J45.100, J45.800, J45.900, J45.902, J45.903, J45.904, J46.x 00); (2) residential addresses of the patients are in urban districts of Shijiazhuang; (3) the age is older than 18 years old.

### Air pollution and meteorological data

Ambient air quality data and meteorological data including daily average temperature and air pressure over the study period were derived from the Hebei Meteorological Bureau, where collects the real-time pollutant concentration from 9 evenly-distributed fixed monitoring stations over urban Shijiazhuang. The average concentration of the nine monitoring stations is taken as the urban pollution level. The daily concentrations of PM_2.5_, PM_10_, SO_2_, NO_2_, CO were calculated as the 24-h mean concentration, while the daily concentration of O_3_ was calculated as the maximum 8-h moving average level.

### Statistical methods

The whole time series was split into two parts in terms of heating season (from November 15 to March 15 of the next year, during which the urban city burned coal for central heating) and non-heatingseason. and all analyses were conducted separately. Quasi-Poisson regression based on generalized additive model combined with penalized distributed lag nonlinear models (DLNMs) for air pollutants and meteorological variables was used to evaluate the associations between daily air pollutants and daily asthma hospitalizations allowing for potentially non-linear and lagged effects [3]. The DLNMs can describe complex nonlinear and lagged dependencies through a cross-basis function, obtained by the combination of two functions that define the traditional exposure-response relationship and the additional lag-response relationship, respectively [8, 9]. Specifically, we primarily selected cross-basis composed of penalized B-splines with the default dimensions of 10 for the two spaces of exposure-response and lag-response. The penalized framework of DLNMs offer build-in model selection procedures, and the penalized B-splines have good performance in multidimensional smoothing [10]. The initial lag day was specified to 7 because previous studies have shown the lagged effect of air pollutants on health is short. The reference exposure concentration of pollutants used for calculating relative risks was set to zero. To avoid the morbidity displacement by “harvesting” which usually occurred in the temporal lagged effect analyses [11, 12], we reselected the lag day according to the maximum lag days that showed no negative effects across the whole range of concentration of pollutants. Then, the linear function for the exposure-response space was specified to obtain the traditional risk estimates for per 10 μg/m^3^ increase in every pollutant(CO: per 0.1 mg/m^3^). Meteorological variables commonly have nonlinear exposure-lag-response associations with respiratory morbidity, and the lagged effects are commonly longer than air pollutants [13, 14]. So the same penalized B-splines were specified both in the two dimensions of DLNMs except the lag period was extended to 14 days for mean daily temperature and air pressure. Long-term and seasonal trend was controlled for using natural cubic splines with 5 degrees of freedom per year according to previous studies. Day of week and statutory holiday were adjusted for as categorical factors.

ANs and AFs were calculated to evaluate the burden for asthma hospitalizations using the method introduced in previous studies based on the cumulative relative risks derived from the non-linear exposure-lag-response models, and the empirical confidence intervals were obtained by Monte Carlo simulations assuming a multivariate normal distribution of the best linear unbiased predictions of coefficients [15]. Cumulative ANs and AFs from 0 to every individual concentration of pollutants in the study period were also calculated. Furthermore, the target levels set by the Chinese National Ambient Air Quality Standards grade II (NAAQS grade II) were considered as a reference to assess the reduction in asthma hospitalizations burden if the air quality level attained NAAQS grade II targets [16].

Multivariate Wald test was used to analyze statistically significant differences of cumulative exposure-response curves between heating and non-heating seasons [8]. The method for calculating *P* value for interaction was used for the comparison of attributable measures between the two seasons [8, 13, 17].

For all statistical tests, statistical significance was defined as *P* < 0.05. All analyses were done using R V.3.4.0 software with “DLNM” and“mgcv”packages.

## Results

Over the 1461 days of the study, there were a total of 1815 and 2230 hospitalizations in heating season and non-heating season respectively. The daily hospitalizations, daily concentrations of PM_2.5_, PM_10_, CO, NO_2_, and SO_2_ were significantly higher while the concentrations of daily O_3_, daily mean temperature and air pressure were significantly lower in heating season than those in non-heating season (Table 1).

**Table 1:** Descriptive statistics of daily number of asthma admissions, meteorological factors and air pollutants in Shijiazhuang, during 2013–2016

	mean	sd	min	25%Q	50%Q	75%Q	max
heating season(604 d)							
daily admissions (*n* = 1815)*	3.00	0.99	0.00	2.00	3.00	4.00	7.00
PM_2.5_(μg/m^3^)*	162.22	120.63	9.00	71.00	128.00	212.14	750.50
PM_10_(μg/m^3^)*	263.16	164.32	18.00	142.75	227.00	349.50	926.00
CO(mg/m^3^)*	2.49	1.75	0.20	1.20	2.00	3.30	12.60
NO_2_(μg/m^3^)*	73.29	31.50	13.00	50.96	68.50	93.00	188.00
SO_2_(μg/m^3^)*	106.49	74.65	4.00	52.00	85.00	137.14	515.43
O_3_(μg/m^3^)*	39.79	27.83	2.00	16.00	34.17	57.00	183.00
daily mean temperature (°C)*	3.66	5.53	−9.40	−0.50	2.90	7.20	20.00
daily mean air pressure (kPa)*	51.52	21.30	12.00	34.00	50.00	68.00	97.00
non-heating season(856 d)							
daily admissions (*n* = 2230)	2.61	0.82	0.00	2.00	3.00	3.00	5.00
PM_2.5_(μg/m^3^)	84.13	60.00	6.00	40.00	71.07	109.00	421.57
PM_10_(μg/m^3^)	164.54	104.23	17.00	85.63	138.00	213.25	679.86
CO(mg/m^3^)	1.00	0.50	0.10	0.70	0.90	1.21	3.91
NO_2_(μg/m^3^)	46.00	20.60	9.00	31.00	43.71	58.08	132.00
SO_2_(μg/m^3^)	34.19	25.97	3.00	16.00	28.00	43.00	178.73
O_3_(μg/m^3^)	109.54	50.20	4.00	76.00	105.00	147.00	262.00
daily mean temperature (°C)	22.25	5.72	3.10	18.60	23.30	26.80	35.50
daily mean air pressure (kPa)	61.42	18.38	12.00	48.00	63.00	75.00	99.00

*:*P* < 0.001 compared with non-heating season using Wilcoxon test

*sd* standard deviation, *Q* quantile

**Fig. 2:**
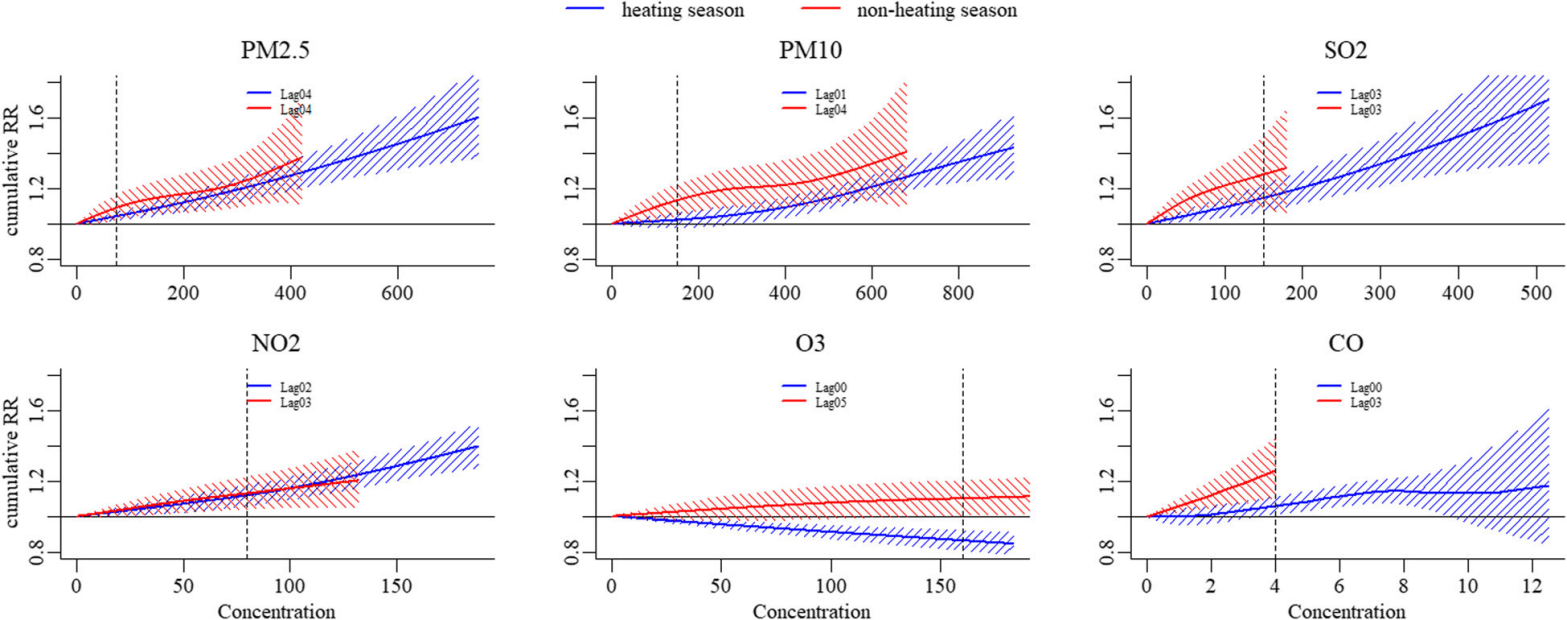
The comparison of cumulative exposure-response associations for pollutants between seasons. Vertical dotted lines: pollutant concentration of Chinese NAAQS grade II

All pollutants demonstrated positive and significant associations with asthma hospitalizations both in heating season and non-heating season, except for O_3_ in heating season where a negative association was observed. The lag period, which representing the maximum lag days without displacement effect, was longer in non-heating season for PM_10_, NO_2_, O_3_, and CO than that in heating season, but there was no difference for PM_2.5_ and SO_2_ between the two seasons. The majority of associations between asthma hospitalizations and pollutants in heating season and non-heating season were approximately linear, while a slightly reversed “S” shaped association for PM_2.5_,PM_10_ in non-heating season and CO in heating season, a slightly “J” shaped association for PM_10_ in heating season and a slightly reversed “J” shaped association for SO_2_ in non-heating season were observed. The differences of overall cumulative exposure-response association between the two seasons were not statistically significant except for O_3_ (*P* values: 0.753, 0.426, 0.939, 0.904, 0.977 and 0.015 for PM_2.5_, PM_10_, SO_2,_ NO_2_, CO and O_3_, respectively), based on the multivariate Wald tests (Fig. 2).

In non-heating season, for the NAAQS grade II target levels vs null exposure, SO_2_(RR:1.28,95% CI:1.094,1.498) and CO (RR:1.26, 95%CI:1.103,1.440) demonstrated the strongest associations with asthma hospitalizations, while in heating season, SO_2_(RR:1.147, 95%CI:1.075,1.224) and NO_2_ (RR:1.118, 95%CI:1.061,1.179) showed the top two strongest associations with asthma hospitalizations. In terms of linear exposure-response models, the top two pollutants with strongest associations with asthma hospitalizations were SO_2_ and NO_2_ both in the two seasons (Table 2).

**Table 2:** The estimates of RR (95%CI) for asthma hospitalizations using different exposure-response functions

	PM_2.5_	PM_10_	SO_2_	NO_2_	CO	O_3_
heating season						
per 10μg/m^3^ increase^a^	1.006	1.003	1.011	1.018	1.002	0.991
	(1.005,1.008)	(1.003,1.004)	(1.008,1.014)	(1.014,1.023)	(1.002,1.003)	(0.987,0.995)
NAAQS grade II^b^	1.043	1.022	1.147	1.118	1.061	0.864
	(1.021,1.066)	(0.981,1.064)	(1.075,1.224)	(1.061,1.179)	(1.014,1.111)	(0.810,0.922)
non-heating season						
per 10μg/m^3^ increase	1.007	1.005	1.02	1.016	1.006	1.006
	(1.004,1.010)	(1.003,1.007)	(1.011,1.029)	(1.007,1.026)	(1.002,1.009)	(1.001,1.010)
NAAQS grade II	1.092	1.133	1.28	1.13	1.26	1.101
	(1.024,1.164)	(1.050,1.222)	(1.094,1.498)	(1.043,1.223)	(1.103,1.440)	(1.007,1.203)

^a^per 0.1 mg/m^3^ for CO and estimated from the linear exposure-response function

^b^estimated from the non-linear exposure-response function versus null exposure

*CI* confidence interval

In heating season,NO_2_,with the overall AN and AF of 182.028(95%CI: 106.111, 257.134) and 10%(95%CI: 5.8, 13.7%) was associated with the heaviest asthma hospitalization burden caused by pollutant exposure, followed by SO_2_, with the overall AN of 169.392(95%CI:97.695, 247.563) and the AF of 9.3%(95%CI:5.2, 12.7%). In non-heating season, the pollutants associated with the top two heaviest burden were PM_10_ and PM_2.5_,with the AN and AF of 252.334(95%CI:121.342, 380.115) and 11.3%(95%CI:4.9, 17%), 186.101(95%CI:73.435, 293.414) and 8.3%(95%CI:3.1, 13.2%), respectively. However, there was no significant difference of attributable measures between heating season and non-heating season, except that the overall AN for PM_10_, from non-heating season was significantly more than that from heating season (*P* = 0.039). and that both the overall AN and AF for O_3_ from non-heating season were higher than that from heating season (AN: *P* = 0.011, AF: *P* = 0.004).

**Fig. 3:**
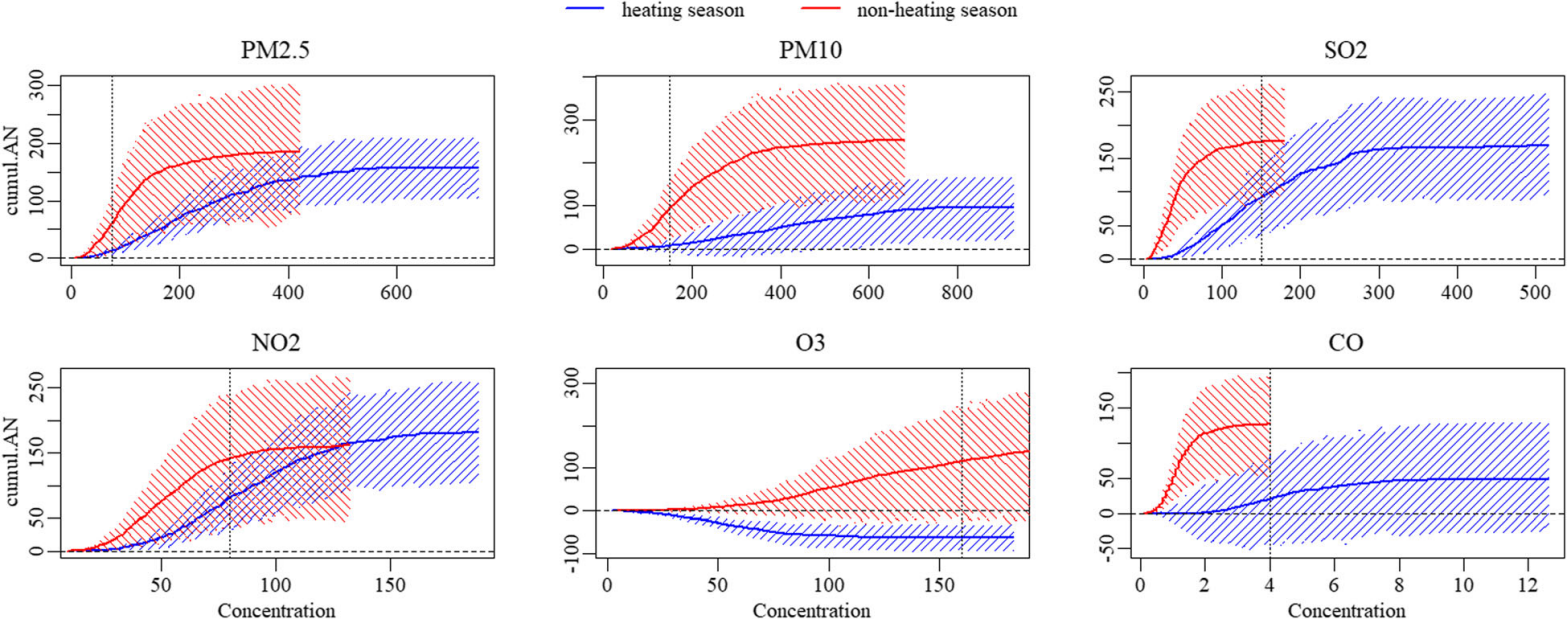
The cumulative attributable number of asthma hospitalizations due to exposure to pollutants in different seasons. Vertical dotted lines: pollutant concentration of Chinese NAAQS grade II

**Fig. 4:**
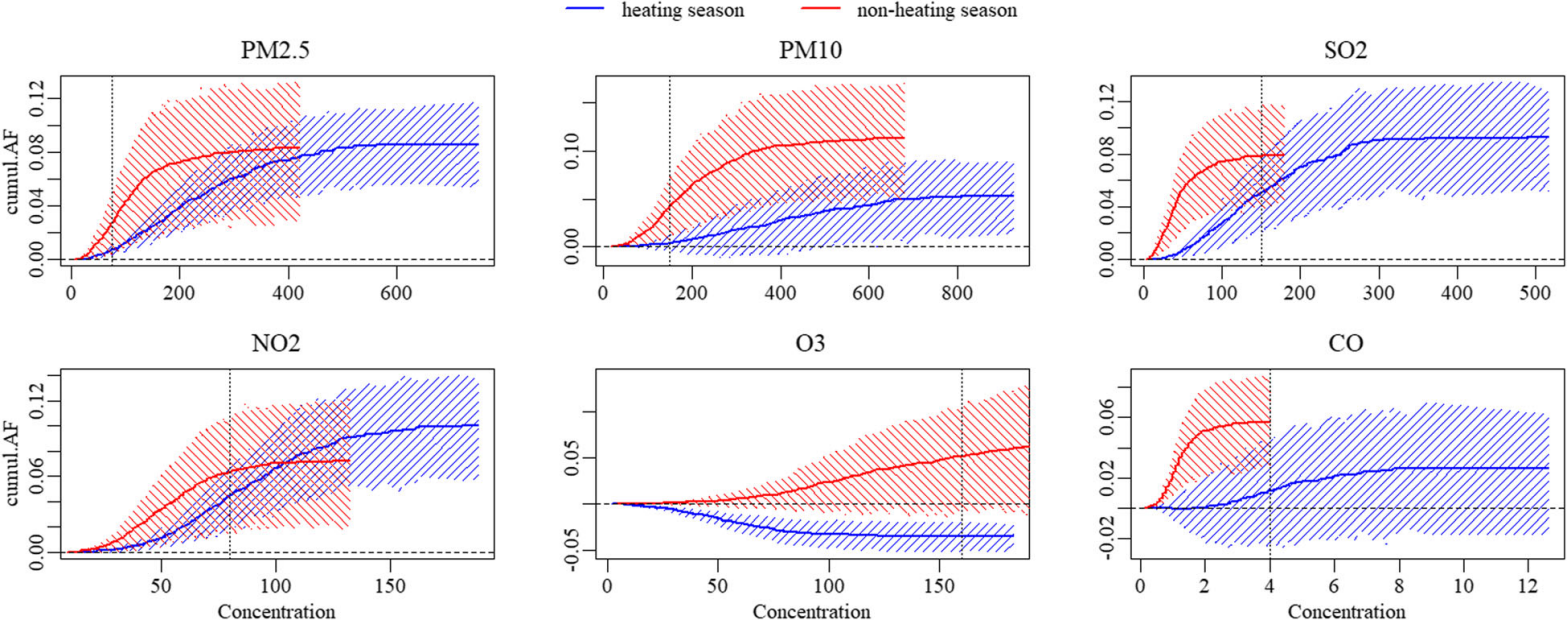
The cumulative attributable fraction of asthma hospitalizations due to exposure to pollutants in different seasons. Vertical dotted lines: pollutant concentration of Chinese NAAQS grade II

If the concentrations of pollutants attained the target levels of NAAQS grade II in heating season, the greatest reduced AN would be 144.247(95%CI: 91.775, 191.827) from PM_2.5_, followed by 101.905(95%CI: 70.140, 137.544) from NO_2_, accounting for about 92 and 56% of the overall AN, respectively. Moreover, a considerable reduction in AF was observed from PM_10_ (decreased AF: 4.9, 95%CI: 1.7, 7.9%), accounting for about 92% of overall AF, though the corresponding reduced AN was only 89.603(95%CI: 30.452, 146.015). As for in non-heating season, the greatest burden reductions come from PM_10_ and PM_2.5_, with decreases in hospitalizations by about 67 and 63%, respectively. There was almost no reduction in asthma hospitalizations for SO_2_ and CO in non-heating season and O_3_ in heating season, because there was no day with CO concentration and only 2 days with SO_2_ and O_3_ concentrations higher than the levels of NAAQS grade II in the corresponding season. Referring to the target levels of NAAQS grade II, the reductions in burden of asthma hospitalizations attributed to pollutants exposure would be significantly higher from heating season for SO_2_, NO_2_, and CO than those from non-heating season. (*P* < 0.001) (Table 3, Figs. 3, 4).

**Table 3:** The comparison of reductions in attributable measures (95%CI) between seasons when various targets attained

			heating season		non-heating season		*P*
**PM**_**2.5**_	NAAQS grade II	AN	144.247	(91.775,191.827)	125.711	(63.306,188.752)	0.651
		AF	0.079	(0.052,0.104)	0.056	(0.026,0.086)	0.256
	NO pollution	AN	156.767	(104.504,208.363)	186.101	(73.435,293.414)	0.636
		AF	0.086	(0.054,0.117)	0.083	(0.031,0.132)	0.921
**PM**_**10**_	NAAQS grade II	AN	89.603	(30.452,146.015)	158.481	(87.590,233.751)	0.147
		AF	0.049	(0.017,0.079)	0.071	(0.040,0.102)	0.325
	NO pollution	AN	96.527	(24.105,166.702)	**252.334**	**(121.342,380.115)**	0.039
		AF	0.053	(0.014,0.089)	0.113	(0.049,0.170)	0.099
**SO**_**2**_	NAAQS grade II	AN	**78.081**	**(50.389,100.071)**	1.098	(0.380,1.665)	<0.001
		AF	**0.043**	**(0.028,0.057)**	0	(0.000,0.001)	<0.001
	NO pollution	AN	169.393	(97.695,247.563)	175.952	(95.114,253.964)	0.906
		AF	0.093	(0.052,0.127)	0.079	(0.045,0.116)	0.595
**NO**_**2**_	NAAQS grade II	AN	**101.905**	**(70.140,137.544)**	20.799	(8.632,33.041)	<0.001
		AF	**0.056**	**(0.037,0.074)**	0.009	(0.004,0.014)	<0.001
	NO pollution	AN	182.028	(106.111,257.134)	161.705	(58.121,264.812)	0.756
		AF	0.1	(0.058,0.137)	0.073	(0.022,0.122)	0.406
**CO**	NAAQS grade II	AN	**28.72**	**(13.043,41.699)**	0	(0.000,0.000)	<0.001
		AF	**0.016**	**(0.007,0.023)**	0	(0.000,0.000)	<0.001
	NO pollution	AN	47.601	(−25.486,129.523)	126.827	(52.925,194.297)	0.139
		AF	0.026	(−0.017,0.062)	0.057	(0.029,0.088)	0.218
**O**_**3**_	NAAQS grade II	AN	−0.779	(−1.165,-0.438)	**39.887**	**(4.494,68.760)**	0.013
		AF	0	(−0.001,0.000)	**0.018**	**(0.002,0.030)**	0.012
	NO pollution	AN	−63.776	(−92.863,-33.397)	**155.081**	**(−17.711,314.930)**	0.011
		AF	−0.035	(− 0.052,-0.022)	**0.07**	**(− 0.003,0.137)**	0.004

*AF* attributable fraction, *AN* attributable number, *CI* confidence interval

Bold: statistically significantly higher than the other season

## Discussion

Although the associations between ambient air pollutants and asthma morbidity have been well documented worldwide, little is known about the burden of asthma hospitalization attributable to air pollution [18–20]. Regarding effect modifier, most previous studies used “cold” and “hot” seasons to assess the seasonal variations of exposure-response associations, while in the present study, we selected heating as a modifier based on relevant environmental studies [7, 21–25]. Furthermore, considering the effect summaries based on ratio measures, such as relative risk or odds ratio, offered less information on the actual impact of the exposure [15], we examined the asthma hospitalization burden due to pollutants exposure using AF and AN in the north China city, Shijiazhuang, where suffers from heavy regional air pollution in the last decades, especially in heating season.

Our study data demonstrated the concentrations of pollutants including PM_2.5_, PM_10_, SO_2_, NO_2_, and CO were significantly higher in heating season than those in non-heating season, except for O_3_ which was a major ambient air pollutant in summer. The concentrations of pollutants were also obviously higher than those in previous studies conducted in other locations.

The hypothesis of effect modification by heating on asthma hospitalizations was reasonable. The concentrations of ambient air pollutants in heating season and non-heating season were significantly different [21]. Existing studies have confirmed that a higher level of air pollution was derived from coal combustion during the local heating period [22]. In winter, coal combustion contributed 26.62% of the particulate matter, which was the most among various sources, while the contribution was only 5.7% in summer [23]. The PM level in heating season can be enhanced by 70% due to coal combustion emissions [24]. Pardo M et al. found urban PM_2.5_ collected in heating season in Beijing, China contained higher levels of pollution components (e.g., metals and polycyclic aromatic hydrocarbons) than that collected in non-heating season. An increased inflammatory response was detected in the lung following exposure to the organic extracts mostly in the PM_2.5_ from heating season [25]. SO_2_ and NO_2_ are two key primary gaseous pollutants emitted by coal burning. The intensity of SO_2_ and NO_2_ emissions are significantly greater during heating season than those during non-heating season [7]. Considering the reasons above, we selected heating period as the effect modifier which would possibly be more appropriate for characterizing the fluctuations of pollutant level than simply classifying the study period into “cold” and “hot” seasons, although there was a considerable overlap between the two classification methods.

Ko FW et al. demonstrated significant associations between asthma hospital admissions and levels of NO_2_, O_3_, PM_10_, and PM_2.5_, with relative risks for every 10 μg/m^3^ increase of 1.028, 1.034, 1.019 and 1.021, respectively, at lag days ranged from 0 to 5 [26]. In the study conducted by Sunyer J et al., daily asthma admissions in adults increased significantly with increasing ambient levels of NO_2_ (RR: 1.029, per 50 μg/m^3^ increase, 95% CI 1.003 to 1.055), while the association between asthma admissions and O_3_ was heterogeneous among cities [27] . Our study confirmed these positive associations between asthma admissions and pollutant exposure in Shijiazhuang, China, both in heating and non-heating seasons. During our study period, the concentrations of pollutants were significantly higher than those in the studies mentioned above especially for PM_10_ and PM_2.5_, and what is more, the concentrations of pollutants in heating season were approximately 2–3 times higher than those in non-heating season except for O_3_. A newly reported investigation conducted in Beijing, the capital city of China and close neighbor of Shijiazhuang, showed every 10 μg/m^3^ increase in PM_2.5_ was significantly associated with a 0.67% (95% CI, 0.53,0.81%), 0.65% (95% CI, 0.51,0.80%), and 0.49%(95% CI, 0.35,0.64%) increase in total hospital visits, outpatient visits and emergency room visits on the same day, respectively, but there was no significant association with hospital admission [28]. Another study conducted in Shanghai, found that a 10 μg/m^3^ increase in the concentrations of SO_2_, CO, NO_2_, PM_10_, O_3_ and PM_2.5_ corresponded to increase of 3.79% (95% CI: 0.84, 6.83%), 0.27% (95% CI: 0.14, 0.40%), 0.63% (95% CI: - 0.81, 2.10%), 1.11% (95% CI: 0.38, 1.85%), 0.23% (95% CI: 0.31, 078%) and 1.27% (95% CI: 0.29, 2.26%) in daily asthma patient visits on the current day [29]. As main mega-cities of China, Beijing and Shanghai have faced the same severe air pollution as well as Shijiazhuang. The differences in, such as the socio-demographic status of residents and the structure of healthcare system might result in the heterogeneity among these studies with similar pollution situations.

In our study, though the associations estimated from either linear or nonlinear exposure-response models both showed stronger associations with asthma hospitalizations in non-heating season, however, no significant differences of the overall exposure-response associations between the two seasons were obtained. A systemic review and meta-analysis including 87 studies reported that associations between pollutants exposure and asthma hospitalizations were stronger in warm season [30]. There are some possible reasons for the higher risks in warm season or non-heating season: First, because the lower pollution level results in fewer competing pollutants, the associations were relatively stronger in non-heating season even if the associations were season-independent. Second, the actual levels of pollutant exposure were biased by the differences between indoor and ambient air pollution levels. Urban residents spent the majority of time indoor, especially in heating season. The concentrations of pollutants indoor were lower than those in ambient air except when ambient concentrations dropped sharply to very low levels, or there were internal emissions from cooking, tobacco smoking or other activities [31]. The difference of indoor and outdoor pollutant level was more significant in heating season. The ambient PM_2.5_ contributed approximately 52 and 42% to indoor PM_2.5_ for non-heating and heating seasons, respectively [32]. The particle sizes indoor were smaller than those in ambient air in the heating season and vice versa in non-heating season [33]. Third, although the cold temperature can stress the body and result in symptom exacerbation, whereas hot temperature is associated with higher asthma prevalence, due to increased exposure to other relevant factors such as allergens [34], rainfall or humidity [35, 36] .

Notably, in spite of the higher level of pollution in heating season, there was no significant difference in the asthma hospitalization burden due to exposure to pollutants between the two seasons, except for total AN for PM_10_ exposure which was significantly higher in non-heating season. The possible interpretations were: First, the calculation of AN and AF was based on the cumulative relative risks (RRs), which was seemingly higher in non-heating season in the present study. Second, significantly skewed distribution of concentration of pollutants, especially in heating season resulted in a relatively less number of days with higher pollutant concentration. The curves of Figs. 3 and 4 illustrated plateaus when the concentrations above approximate 400 μg/m^3^, 600 μg/m^3^, 270 μg/m^3^, 140 μg/m^3^ and 6 mg/m^3^ for PM_2.5_, PM_10_ SO_2_, NO_2_ and CO in heating season, respectively, indicating that beyond these cutoff values, the increment of asthma hospitalization burden was negligible. However, the corresponding cutoffs (i.e., approximate 200 μg/m^3^, 400 μg/m^3^, 100 μg/m^3^, 80 μg/m^3^ and 2 mg/m^3^ for PM_2.5_, PM_10_ SO_2_, NO_2_ and CO, respectively) were about 50% lower and the plateaus were shorter for non-heating season compared with those for heating season. These results were reasonable and consistent with the higher relative risks for pollutants in non-heating season considering the distinctly skewed distributions of pollutant concentrations, especially in heating season.

It is a standard practice for countries to release Ambient Air Quality Standards, so as to set legal limitations upon the amount of pollution released into the environment [1]. We adopted the NAAQS of China grade II for a reference, which was used in previous studies [16, 37], to evaluate its capability to protect people with asthma. In our study, the burden of asthma hospitalizations due to exposure to pollutants exceeding the levels of NAAQS grade II in heating season accounted for approximate 92,92,46,56,61 and 0% of the overall burdens due to PM_2.5_, PM_10_,SO_2_, NO_2_, CO and O_3_ exposure, respectively. In non-heating season, the corresponding proportions were about 67,62,0,12,0 and 26%.I f the target of NAAQS grade II for PM_2.5_, PM_10_, SO_2_, NO_2_ and CO are achieved in heating season, most of the asthma hospitalizations related to air pollution would be reduced, while in non-heating season, only the hospitalizations caused by PM_2.5_ and PM_10_ exposure could achieve considerable reductions. This suggests that part of the current standards may be inadequate to protect asthma patients and benefits can be achieved from the continuous reduction of air pollution, even if air quality attained the NAAQS grade II levels especially for SO_2_, NO_2_, O_3_ and CO in non-heating season.

There are two other aspects worth to be noticed in our study results: First, although SO_2_ and NO_2_ showed the strongest associations with asthma hospitalizations in linear exposure-response models both in two seasons and the highest attributable measures in heating season, however, in non-heating season, the top two pollutants related to the heaviest burden were PM_10_ and PM_2.5_.This was mainly because compared to other pollutants, SO_2_ and NO_2_ had more days with higher level as well as higher RRs in heating season. In contrast, there were more days with higher PM_10_ and PM_2.5_ levels than SO_2_ and NO_2_ in non-heating season. Second, the significantly negative association between O_3_ and asthma hospitalizations in heating season possibly due to the potential confounders with O_3_ existing in heating season that had not be included in our investigation.

### Strength

This study focused on the entirely urban population of Shijiazhuang city, one of the most severely polluted cities in north China, where the air pollutant mixtures and meteorological factors may potentially be quite different due to the unique topography.

Also, we used a flexible statistical approach (i.e., a penalized framework of distributed lag non-linear model) to investigate the exposure-lag-response association between air pollution and asthma hospitalizations and further analyzed the burden due to pollutants exposure. To our knowledge, this is the first time to apply the newly-presented statistical approach in respiratory disease study.

Furthermore, we evaluated potential effect modification by heating, which was investigated as an effect modifier for the first time as well.

### Limitations

Potential limitations should be taken into consideration.

First, outdoor fixed monitoring stations resulted in unavoidable individuals’ exposure measurement error, which is an inherent limitation of epidemiology studies of disease and environment.

Second, to avoid the unstable estimates because of the concurvity between pollutants, this study used single pollutant models. Although many studies of the health effects of air pollution focused on individual pollutants, actual air pollution exposures are usually multipollutant mixtures [38], so the results of this study cannot represent the independent effects of pollutants due to the presence of confounding between them.

Third, we did not examining the effect modification of individual characteristics to identify vulnerable subgroups because of the relatively less daily counts of hospitalizations. Individual-level data such as gender, age, smoking, activity patterns and indoor pollutants exposure should be further studied.

## Conclusion

In conclusion, we found evidence of the effect of ambient air pollutants on asthma hospitalizations in Shijiazhuang. SO_2_ and NO_2_ showed the strongest associations with asthma hospitalizations both in heating season and non-heating season, however, the differences between the two seasons were not significant. Meanwhile, SO_2_ and NO_2_ caused the heaviest burden of asthma hospitalizations in heating season, but in non-heating season, PM_10_ and PM_2.5_ were responsible for the heaviest burden. A better understanding of these associations between ambient air pollutants and asthma hospitalizations has important public health implications for planning intervention measures to reduce the risk of asthma hospitalization burden, especially when taking the effect modification by heating into consideration.

## Data Availability

Data can be available through contact with the corresponding author.

## Abbreviations

AF: attributable fraction
AN: attributable number
CI: Confidence interval
CO: Carbon monoxide
DLNMs: distributed lag nonlinear models
GAM: Generalized additive model
NAAQS grade II: national ambient air quality standards grade II
NO_2_: Nitrogen dioxide
O_3_: Ozone
PM_10_: Particle matter<10 μm in diameter
PM_2.5_: Particle matter<2.5 μm in diameter
Q: quantile
sd: standard deviation
SO_2_: Sulfur dioxide
